# Events associated with stability and change in adult locus of control orientation over a six-year period

**DOI:** 10.1016/j.paid.2018.01.017

**Published:** 2018-05-01

**Authors:** Stephen Nowicki, Genette Ellis, Yasmin Iles-Caven, Steven Gregory, Jean Golding

**Affiliations:** a Department of Psychology, Suite 270, PAIS Building, 36 Eagle Row, Atlanta, GA 30322, USA; b Centre for Child and Adolescent Health, Bristol Medical School, University of Bristol, Oakfield House, Oakfield Grove, Bristol BS8 2BN, UK

**Keywords:** ALSPAC, Parental locus of control, Longitudinal cohort, Stability over time, Life stressors

## Abstract

Although locus of control (LOC) has been the focus of thousands of studies we know little about how or if it changes over time and what is associated with change. Our lack of knowledge stems in part from the past use of cross-sectional and not longitudinal methodologies to study small numbers of participants from non-representative populations. The purpose of the present study was to use a longitudinal design with a large representative population to provide relevant information concerning the stability and change of adult LOC. Before the birth of their child, and again six years later, mothers and their partners participating in the Avon Longitudinal Study of Parents and Children (ALSPAC) completed LOC tests and structured stressful events surveys. Analyses revealed that stresses experienced in relationships with spouses, friends and family, financial stability and job security, and illness/smoking were associated with changes in LOC. Results suggest substantial variation of LOC within spousal/parent dyads and moderate stability of LOC over time for both men and women. Stressors associated with change in LOC may be possible candidates when considering interventions to modify LOC expectancies.

## Highlights

• Little is known as to factors associated with changes in LOC over time.
• This is a unique data set measuring LOC in men and women over a 6 year period.
• Results show relationship stressors were associated with change in LOC orientation.
• Stressors could be candidates when considering interventions to modify LOC.
• Substantial variation of LOC within spousal dyads was found.

## 1 Introduction

The purpose of this project was to examine the stability and change of locus of control (LOC) orientation in adult men and women over a 6 year period, and to identify events associated with LOC stability or change. LOC refers to individuals' generalized expectancy regarding the connection between their behavior and reinforcements received in a problem-solving context (Rotter, 1966).

Individuals who fail to see a connection between what they do and what happens to them and view what happens as the result of luck, fate, chance, or powerful others are *externally controlled*. Conversely, those who tend to perceive a connection between their efforts and what happens to them are *internally controlled*.

Rotter's article stimulated a remarkable amount of research. A search of PsychInfo resulted in 17,812 articles with a keyword “locus of control” as of summer 2015 and with 6600 of these appearing after 1996 (1425 dated 2010–2015). LOC has sustained itself as a concept for psychological study for more than a half century (Nowicki & Duke, 2016).

As there are >100 different definitions of “locus of control” throughout the literature (Skinner, 1996), researchers need to clearly state and define which LOC concept and measure is being used (e.g. Reich & Infurna, 2016). Peterson and Stunkard (1992) have noted potential problems that could result from using similar appearing cognates, like *efficacy* (e.g. Infurna & Mayer, 2015; Lachman & Weaver, 1998) or *attribution* (Peterson & Seligman, 1983; Seligman, 1975) interchangeably with *locus of control of reinforcement* as described by Rotter (1966). Rotter, 1954, Rotter, 1966 emphasizing that it is an expectancy that has the capacity to affect behavior differently from situation to situation and has its greatest impact in circumstances that are novel, ambiguous or transitory.

LOC has been related to an ever-growing number of important and significant aspects of human life including *personality characteristics* (e.g. Judge & Bono, 2001; Nowicki & Duke, 1974), *social adjustment difficulties* (e.g., Cheng, Cheung, Chio, & Chan, 2013), *academic achievement* (e.g. Flouri, 2006), *health outcomes* (e.g. Conell-Price & Jamison, 2015), and *business success* (e.g. Kormanik & Rocco, 2009). However, surprisingly little research has been completed concerning the origins of control orientations or their trajectories over time.

Using data gathered from the Avon Longitudinal Study of Parents and Children (ALSPAC), Golding, Iles-Caven, Gregory, and Nowicki (2017), Golding, Gregory, Iles-Caven, and Nowicki (2017), and Golding, Ellis, Iles-Caven, Gregory, and Nowicki (2017), identified antecedent characteristics of the childhoods that were common to internality in both men and women; these included maternal warmth, being breast fed, having a stable home, and recollection of childhood as happy. Antecedents like these describe a home situation in which children feel comfortable and safe enough to explore their environments and learn more about the possible contingencies existing between their behavior and outcomes. Lefcourt (1976) had earlier theorized a similar set of circumstances underlying the development of appropriate internal control expectancies.

Antecedent information may be helpful in suggesting what might underlie the learning of generalized internal or external control expectancies as well as to offer possible insights into what may be associated with changes in or maintenance of LOC. Unfortunately, since most of the antecedent data have been obtained from studies using cross-sectional methodologies they are inappropriate for objectively describing how individuals' LOC changes over time or, if changes do take place, with what they are associated. Longitudinal data would be valuable in supporting or refuting cross-sectional results describing the development of control expectancies. The purpose of this investigation was to identify events associated with LOC changes over time by analyzing longitudinal LOC data gathered over a 6 year period beginning when the women and their partners were expecting a baby.

Some LOC studies with adults have used LOC pre-post scores as an outcome measure to evaluate the impact of interventions. Unfortunately, in these cases researchers rarely report correlations between pre-post testing of the group not receiving the intervention, as this would be valuable in determining the stability of LOC over time in typical participants. For example, Sørlie and Sexton (2004) studied the effects of psychosocial medical and treatment related factors upon change in the Multidimensional Health Locus of Control scale (MHLC) (Wallston, Wallston, & DeVellis, 1978). The MHLC was administered to surgical patients prior to surgery and again four months following discharge. They found a positive relationship with physicians' predicted internality, while severity of illness and subjective feelings of stress were associated with externality. Similar findings were obtained with patients who had undergone heart surgery (Sørlie & Sexton, 2004). They too became more internal with the passage of time and involvement with rehabilitation.

Though researchers using the perceived control construct have produced results consistent with those found by Rotter, orientated LOC investigators have primarily studied older rather than younger adults. They used a variety of ways to assess “perceived” control, and not offered construct validity evidence in support of the perceived control measures they used or how they may have related to LOC. For example, in one study (Turiano, Chapman, Agrigoroaei, Infurna & Lachman, 2014) “perceived” control was measured using a 4-item test with a 7-point Likert Scale while in another (Infurna & Okun, 2015) it was assessed by a 6-item scale with a 4-point Likert scale. Because there is an absence of information of how the different perceived control measures correlate with one another or with Rotter's scale, it is difficult to determine if what they are measuring is similar to or different from what researchers have found using Rotter-based LOC scales.

Rather than assessing “perceived” control in middle-aged and older adults, Schneewind (1997) was one of the few researchers to gather test–retest information from younger adults using a scale based on Rotter's definition. The initial test was administered to parents when their child was 10 years old; the retest took place 16 years later. Parents and their children completed German adaptations of LOC scales (Adult and Child Nowicki-Strickland Internal-External scales). LOC was part of a more comprehensive longitudinal study of personality in the context of family development (Schneewind, Ruppert, & Harrow, 1998). Initial data were gathered from a sample of mother–father-child triads recruited from six different German states. The mean age of the children was 12, mothers 39, and fathers 42. Sixteen years later Schneewind and his colleagues contacted former participants and had 197 triads volunteer to participate in a second assessment.

In this paper we are primarily interested in what Schneewind found regarding parent LOC. At both testing times, mother and father LOC scores were positively correlated, but low (time one, r = 0.19; time two, r = 0.21). Pre-post LOC correlations for mothers and fathers across the 16 years were what Schneewind called “moderate” and ranged between 0.35 and 0.44.

### 1.1 The present study

Little is known about how LOC expectancies develop and change in adulthood. We lack information about what the LOC association is within spousal or parental dyads, the trajectory of adult LOC over time or what life events are associated with LOC changes over time. Our goal here is to provide such LOC information.

The ALSPAC data set is unique in that it contains LOC scores from both mothers and fathers *before* the child was born and six years later. Thus we can assess the stability of parents' LOC during a critical period of their lives. The absence of previous research made prediction difficult. Relationship theories offer competing predictions. Complementary theorists (e.g., Kiesler, 1982) suggest a negative association between mother and father LOC scores, with one individual being external and the other internal. Similarity perspectives (e.g., Byrne, 1969) suggests that the LOC scores would be similar; internals liking internals and externals liking externals. Based on Schneewind's findings, we predict parents' own pre-post LOC scores would be moderately related to one another over time, but relatively unrelated to one another at pre- and post-times.

Although Schneewind obtained valuable data regarding adult LOC stability and change, he failed to provide any information about what might be associated with changes. The ALSPAC data set includes data concerning the stressors encountered by adults during the six years between the first and second LOC administration. Rotter (1966) and Lefcourt (1976) theorize that internality thrives when individuals are in warm, supportive, relatively stress free environments in which they can learn to perceive the connection between their behavior and outcomes. We predict that greater stress will be related to greater externality.

## 2 Material and methods

### 2.1 The ALSPAC study

This pre-birth cohort was designed to determine the environmental and genetic factors that were associated with health and development of children and their parents (Golding and ALSPAC Study Team, 2004; Boyd et al., 2013). As part of the study design, and in order to determine the parents' backgrounds prior to the birth of the child, there was a concerted effort to obtain details of their personalities, moods and attitudes, including a measure of their LOC, before the birth of the child.

ALSPAC recruited 14,541 pregnant women resident in Avon, UK with expected dates of delivery 1st April 1991 to 31st December 1992. Enrolment strategies included encouragement through the local media, general practitioners, midwives, health services and obstetric hospitals; women then contacted the study center for further information; they were then sent a series of questionnaires to be completed at home. 14,541 is the initial number of pregnancies for which the mother enrolled in the ALSPAC study. Of these there were 14,062 livebirths, of which 13,988 survived to at least 12 months. For full details of all the data collected see the study website: www.bristol.ac.uk/alspac/researchers/data-access/data-dictionary/.

Uniquely among the major UK cohort studies at the time it was decided to include the fathers of the children. To this end questionnaires were sent to the mother to pass to her partner if she was happy for him to take part. This strategy was approved by the ALSPAC Ethics and Law Committee (Birmingham, 2018). Consequently, there was no immediate way in which the study administrators knew the identity of the study fathers. Given the uncertainty of this approach, it is striking how many took part during the pregnancy (10,000 compared with 13,867 pregnant women (Fraser et al., 2013)).

Follow-up used a variety of techniques including questionnaires, hands-on examinations, assays of biological samples and details of the environment at various stages of life. Questionnaires included detailed history of events, occupations and life styles of each parent. Loss to follow-up occurred when the child died, the mother refused, or moved away and could not be traced. As in all longitudinal studies, attrition increased as the study continued (Fraser et al., 2013). This is illustrated by the numbers of parents who completed the LOC questions at the two time points 6 years apart. For the women the numbers fell from 10,565 to 8378 (79% of the original), and for the partners the change was from 7365 to 3891 (53%). From Supplementary Table 1 it can be seen that the proportion of parents with LOC scores 6 years after the study child's birth had proportionately fewer families who (a) resided in public housing, (b) were young mothers, (c) were of manual social class or (d) who smoked. These factors need to be born in mind when generalizing the results.

### 2.2 Measures of LOC

The LOC measure used in the present study is a shortened form of the adult version of the Nowicki-Strickland Internal-External locus of control scale (ANSIE) which comprises 40 items in a yes/no format to assess perceived control (Nowicki & Duke, 1974). This was chosen over other scales more specifically related to perceived control over health, as it was considered that this more generalized scale would relate to other factors in addition to health outcomes. Construct validity for the scale has been found in the results of over 1000 studies (Nowicki, 2016). The version used here comprises 12 of the original 40 items, which were chosen after factor analysis of the ANSIE in a pilot of 135 mothers in the USA. An Anglicized version of these 12 items was included in the questionnaires sent to the two parents in pregnancy (Golding, Iles-Caven, et al., 2017) and again six years after the child was born, with identical wording. From the responses LOC scores were derived for men as well as for women, the higher the score the more external the LOC. The scores ranged from 0 to 12.

Classification into external and internal parents in pregnancy, using the definition for externality of greater than the median LOC score (>4 for women, >3 for men), identified 40.3% of mothers and 38.8% of their partners as externally-oriented in pregnancy. Because of increasing internality in the women over time, their median LOC score changed to 3, whereas their partners stayed at 3.

### 2.3 Identification of events

From 8 months post-birth, each parent was sent a questionnaire concerned with life events in the preceding period, at approximately yearly intervals. The life events inventory comprised 42 items, which were derived for the ALSPAC study using previous inventories as a basis for selection of items. Three main sources were used in this way: Brown and Harris (1978), Barnett, Hanna, and Parker (1983) and Stanley (1988).

In order to develop a summary of variables to determine whether each specific event had occurred over the preschool period of the study child, we created a variable for each event that used all available questions (i.e. those sent to the parents at 8, 21, 33 and 47 months). We did not sum the number of times an event occurred as there was often overlap between ages. Rather than look at the number of the events occurring, we have deliberately looked at each separately using the hypothesis that some types of event will result in increasing internality, and some externality, a pattern that we found when considering the relationship between the events in childhood and the LOC of the adult (Golding, Ellis, et al., 2017).

### 2.4 Other variables considered

Other variables investigated here were chosen because they were associated with parental LOC as well as with differential response rates. They were included to determine whether they were associated with any change in LOC orientation across the 6 years. They include: (1) housing tenure – which is an accepted socio-economic indicator in the UK – divided into owner-occupied (includes having a mortgage), rented public housing, and other rented property; (2) age of the mother at the child's conception; (3) social class (based on occupation of the mother's partner classified into manual and non-manual (Standard Occupational Classification, 1990); (4) presence of mother's partner in the home (identified by questionnaire at 8 months); (5) maternal education level (based on the actual qualifications attained and divided into three levels of attainment); (6) the mother's parity (number of previous pregnancies resulting in either a live or stillbirth); (7) the smoking habit of each parent at 8 months of age (categorized in two ways – any regular smoking and regular smoking of ≥10 cigarettes/day).

### 2.5 Statistical techniques

The statistical strategy was to determine the pattern of factors that were associated with the change in LOC orientation in each parent, with a focus on events that occurred after the initial measure. The study was hypothesis free. Based on the findings from a study of the childhoods of these individuals we anticipated that some of the events might result in a change toward internality, and some toward externality. Results were descriptive, and compared individuals who changed from those who did not, using logistic regression. No account was taken of the number of tests undertaken, to avoid type I errors.

## 3 Results

### 3.1 Correlation of parents' LOC scores with one another and across time

As demonstrated in Table 1, both mother and father LOC scores were positively and moderately correlated between the two-time points (mothers = 0.57; fathers = 0.56); cross-sectional correlations between parents at each of the two time points were positively correlated, but low (in pregnancy r = 0.33; 6 years later r = 0.29).

**Table 1:** Correlation between mother's locus of control in pregnancy, partner's locus of control in pregnancy, mother's locus of control at 6 years and partner's locus of control at 6 years (N = 3887).

	Mother in pregnancy	Father in pregnancy	Mother 6 years later	Father 6 years later
Mother in pregnancy	1.000			
Father in pregnancy	0.331	1.000		
Mother 6 years later	0.570	0.244	1.000	
Father 6 years later	0.290	0.558	0.292	1.000

### 3.2 Additional findings concerning parents' LOC

The ‘change score’ was calculated as the difference between the LOC score in pregnancy and that obtained 6 years later. Mothers' and fathers' LOC change scores ranged from −9 to +8, with mode and median at 0 with a mean difference of −0.30 [SD 1.89; n = 8302] points for mothers and +0.037 [SD 1.98; n = 3968] points for fathers. Thus, mothers on average were getting more internal while their partners were tending to become more external. The correlation coefficient between the parents change scores was positive but low (0.11).

### 3.3 Lifestyle and social conditions associated with changes in LOC over time

In Table 2a, Table 2b we consider certain *lifestyles* and *social conditions* at 8 months post- delivery: for life-style we consider the changes in LOC associated with the *smoking habits* of each parent; social conditions were demonstrated by the *tenure of the home* in which the family were living and whether the mothers' *partner was part of the household*.

**Table 2a:** Variation in social and lifestyle circumstances at 8 months associated with change in maternal LOC orientation from pregnancy to 6 years later.

Social and lifestyle circumstances at 8 m	Mother stayed external	Mother became internal	P	Mother became external	Mother stayed internal	P
	%	%		%	%	
Parental smoking						
Mother smoked	30.7	24.7	.002	20.0	11.9	<.0001
Mother smoked 10+ cigarettes per day	21.7	15.8	<.001	12.4	6.0	<.0001
Partner smoked	33.8	28.9	.018	25.0	18.2	<.0001
Partner smoked 10+ cigarettes per day	27.4	22.4	.009	19.0	12.6	<.0001

Housing tenure						
Owner-occupied	72.1	80.5		84.9	89.6	
Council	18.0	9.6		8.3	3.4	
Other rented	9.9	9.9	.002	6.9	7.0	.005

Had live-in partner						
Yes	91.3	93.6	.047	95.6	97.4	<.001

[Percentages concern the proportion of women in the LOC category who had the history].

**Table 2b:** Variation in social and lifestyle circumstances at 8 months associated with change in partners' LOC orientation from pregnancy to 6 years later.

Social and lifestyle circumstances at 8 m	Partner stayed external	Partner became internal	P	Partner became external	Partner stayed internal	P
	%	%		%	%	
Parental smoking						
Mother smoked	27.4	16.9	<.0001	18.9	9.4	<.0001
Mother smoked 10+ cigarettes per day	18.6	9.0	<.0001	11.0	4.9	<.0001
Partner smoked	33.2	22.4	<.0001	22.3	12.6	<.0001
Partner smoked 10+ cigarettes per day	26.6	16.7	<.0001	16.2	8.0	<.0001

Housing tenure						
Owner-occupied	77.4	86.3		89.7	93.4	
Council	15.8	6.6		4.1	1.2	
Other rented	6.8	7.0	.007	6.2	5.5	<.001

Had live-in partner						
Yes	97.6	98.3	.362	98.9	99.7	.044

[Percentages concern the proportion of men in the LOC category who had the history].

Smoking of the parents bore very strong associations with the likelihood of the parents changing their orientation (Table 2a). Of the women who *stayed externally oriented* 30.7% were smoking when the offspring was 8 months old, whereas 24.7% of those who had *become internal* had this history (P = .002); for women who *stayed internal*, only 11.9% smoked, but 20.0% of those who *became external* did so (P < .0001). Similar patterns were shown for women who were heavy smokers as well as when the partner smoked (Table 2a). Changes in the partners' orientation were equally associated with the smoking habits of the woman as well as of the partner himself (Table 2b).

Mothers who lived in *public housing* were considerably more likely to stay external, and those in *owner-occupied accommodation* less likely to do so. Conversely mothers who were internally oriented in pregnancy were more likely to become external if *in public housing* (Table 2a). A similar pattern was shown for the mothers' partners (Table 2b).

Most of the women who answered the relevant questionnaires had live-in partners (Table 2a), but those mothers who were *externally oriented* were more likely to *become internal* if the partner was present. Conversely the women who were internal but *became external* were less likely to have a live-in partner. Partners were less likely to be given (or complete) a questionnaire if they were not living with the mother 6 years post-birth; even so, among those who answered the questionnaire, there was some evidence that those who *became internal* were more likely to be living with the mother, and those who did not were more likely to *become external* (Table 2b).

### 3.4 Stressful life events associated with changes in LOC over time

Table 3a presents variations in the potentially stressful events occurring to parents and ways in which they were associated with maternal changes in the LOC orientation. The first two columns compare proportions of women who had a history of the stressor between those who stayed external and those who became internally-oriented. Of the 42 stressors occurring, just *four* showed a significant association: the women were more likely to *become internal* if (a) they went back to work and/or (b) they had problems at work; they were more likely to *stay external* if they had (i) separated from a partner, or (ii) argued with family or friends.

**Table 3a:** Variation in the occurrence of stressors as recorded by the mother between 8 months and 4 years post-delivery associated with changes of LOC over 6 years from pregnancy.

Stressors between 8 and 21 m [mother's report]	Mother stayed external	Mother became internal	P	Mother became external	Mother stayed internal	P
	%	%		%	%	
Death of friend or relative	56.1	55.4	.748	59.6	57.0	.080
Child was ill	65.6	65.2	.852	68.7	69.1	.762
Partner was ill	38.7	41.0	.243	42.6	48.1	<.**001**
Friend or relative was ill	52.2	55.4	.117	56.6	58.7	.153
Mother was hospitalized	38.9	39.5	.757	39.4	41.4	.181
Mother divorced	4.9	4.1	.306	4.8	1.9	<.**0001**
Partner rejected the child	4.6	3.3	.121	3.5	1.6	<.**0001**
Mother was ill	23.2	22.0	.458	21.1	18.1	.**011**
Partner lost job	21.1	19.3	.293	19.5	16.6	.**010**
Partner had problems at work	43.0	46.8	.**063**	48.1	52.7	.**002**
Mother had problems at work	19.4	24.4	.**002**	25.3	30.6	<.**0001**
Mother lost her job	9.1	9.8	.550	9.2	10.4	.190
Partner went away	25.9	25.1	.674	27.0	27.3	.833
Partner in trouble with the law	5.2	6.3	.240	4.3	2.6	.**001**
Mother in trouble with the law	2.1	1.3	.160	1.7	0.9	.**015**
Mother convicted of an offence	1.1	0.8	.482	1.3	0.7	.**026**
Separated from partner	17.5	12.8	.**002**	13.1	6.5	<.**0001**
Mother's income was reduced	63.2	63.4	.926	64.0	64.1	.977
Mother and partner argued	83.0	83.0	.980	82.1	79.5	.**024**
Mother argued with family and friends	42.0	37.1	.**016**	39.1	31.7	<.**0001**
Moved home	40.6	39.6	.615	38.1	38.8	.618
Physically abused by partner	7.8	7.8	.987	6.8	3.8	<.**0001**
Became homeless	3.3	3.8	.545	2.6	1.1	<.**0001**
Major financial problems	34.3	31.4	.128	29.8	25.1	<.**001**
Got married	7.2	5.8	.172	5.2	3.4	.**002**
Started new job	38.9	39.2	.858	39.5	40.7	.397
Went back to work	45.4	51.2	.**004**	53.4	60.2	<.**0001**
Took an exam	13.2	15.8	.061	15.4	16.8	.179
Emotionally abused by partner	22.0	22.7	.670	21.1	14.0	<.**0001**
Partner emotionally abused children	5.7	5.0	.450	4.7	2.8	<.**001**
Mother emotionally abused her children	4.7	4.4	.752	5.4	4.0	.**032**
Mother physically abused children	1.8	2.2	.501	1.8	2.0	.597
Partner physically abused children	1.2	1.2	.910	1.3	0.8	.151
Mother's home/car burgled	30.1	33.6	.070	33.5	36.7	.**024**
Mother attempted suicide	1.2	0.5	.136	1.2	0.2	<.**001**
Partner started new job	31.0	34.9	.**040**	35.1	38.5	.**018**
Pet died	28.8	27.5	.478	27.5	24.9	.**046**
Mother had an accident	8.4	8.4	.955	10.8	11.1	.724
Mother had a miscarriage	8.4	8.2	.911	8.7	9.0	.772
Mother had a termination	3.1	2.7	.608	2.9	2.1	.080
Numbers studied	2607	765		1813	3193	

[Percentages concern the proportion of women in the LOC category who had the history].

In bold are P values of P < .05.

The last two columns compare the occurrence of the events among those who *stayed internal* and those who were internal but *became external*. In this case, *25* of the 42 stressors were significant at the 5% level or higher, with *16* of the 25 at the .005 level and *9* of the 25 at the <.001 levels or better. We find 4 of the 16 significant events associated with *staying internal*: (i) having a partner who was very ill, (ii) having a partner who had problems at work, (iii) mother returning to work, and (iv) having problems at work. In contrast, three times as many stressful events, 12 in all, were associated with *becoming external*: (a) becoming divorced, (b) having a partner who rejected the child, (c) having a partner who was in trouble with the law, (d) separating from the partner, (e) arguing with family and friends, (f) becoming homeless, (g) having major financial problems, (h) getting married, (i) being physically abused by her partner, (j) being emotionally abused by her partner, (k) having a partner who emotionally abused her children, and (l) attempting suicide.

As was found for mothers, partners had more stressful events associated with externality than internality (Table 3b). Only one event was significantly associated with changing from an *external to an internal orientation:* starting a new job. Four were associated with him being significantly less likely to become internal; (a) losing his job, (b) arguing with his partner, (c) arguing with family and friends, and (d) having major financial problems. Similar to the mothers, partners had more stressors, 11 in all, associated with *becoming external*. Four of the 11, were associated with being *less likely to become external* – (a) a friend or relative was ill, (b) he moved home, (c) he had a burglary, and (d) his partner became pregnant. Seven were associated with *becoming external*; (i) having been very ill himself, (ii) losing his job, (iii) reduction in his income, (iv) arguing with family and friends, (v) having major financial problems, (vi) being emotionally abused by his partner, and (vii) his partner starting a new job.

**Table 3b:** Variation in the occurrence of stressors as recorded by the mothers' partner between 8 months and 4 years post-delivery associated with changes of LOC over 6 years from pregnancy.

Stressors between 8 and 21 m [father's report]	Father stayed external	Father became internal	P	Father became external	Father stayed internal	P
	%	%		%	%	
Death of friend or relative	60.5	61.5	.704	58.3	61.0	.252
Child was ill	71.4	73.2	.474	77.9	78.3	.870
Partner was ill	61.2	63.2	.458	65.7	69.3	.101
Friend or relative was ill	54.6	57.2	.348	57.6	63.0	.**020**
Was hospitalized	18.6	16.0	.228	15.9	14.6	.423
Was divorced	1.4	0.6	.208	1.2	0.7	.188
Partner rejected the child	1.2	0.4	.168	0.9	0.9	.999
Was very ill	16.6	15.6	.633	16.6	13.1	.**036**
His partner lost job	7.1	5.4	.293	8.4	6.1	.052
Partner had problems at work	25.2	29.9	.052	28.5	27.5	.642
Had problems at work	59.8	63.0	.241	65.0	67.4	.280
Lost his job	20.2	15.0	.**015**	16.5	11.4	.**002**
Partner went away	11.3	9.1	.217	9.6	11.0	.353
Partner in trouble with the law	1.4	0.6	.208	1.1	0.6	.271
In trouble with the law	7.1	5.0	.121	4.6	4.7	.920
Convicted of an offence	4.2	2.5	.098	3.0	3.3	.669
Separated from partner	4.5	3.7	.479	3.9	2.4	.068
Income was reduced	53.0	47.8	.063	47.8	37.8	<.**0001**
Argued with partner	85.2	81.1	.**043**	82.7	80.0	.162
Argued with family and friends	41.8	33.3	.**002**	39.2	28.5	<.**0001**
Moved home	33.4	35.1	.516	36.3	41.0	.**043**
Physically abused by partner	4.3	5.4	.363	3.7	2.8	.315
Became homeless	1.3	0.8	.446	1.2	0.6	.140
Major financial problems	37.2	26.8	<.**0001**	30.8	21.2	<.**0001**
Got married	5.4	5.4	.988	3.0	2.5	.501
Started new job	34.7	41.2	.**016**	42.9	47.3	.069
Went back to work	5.8	4.1	.187	3.8	3.0	.385
Took an exam	23.3	21.8	.534	23.6	25.9	.279
Emotionally abused by partner	17.5	15.6	.670	17.7	14.0	.**032**
Emotionally abused children	4.8	4.3	.699	3.7	4.3	.499
Partner emotionally abused her children	4.1	2.3	.075	3.0	4.4	.127
Partner physically abused her children	1.1	0.8	.647	0.9	0.9	.904
Physically abused his children	1.6	1.9	.680	1.8	2.4	.394
Home/car burgled	32.8	30.4	.336	35.4	40.0	.**047**
Attempted suicide	–	–	–	–	–	–
Partner started new job	33.9	37.0	.243	40.3	34.2	.**008**
Pet died	28.7	26.4	.356	25.6	26.8	.567
Had an accident	16.5	19.4	.175	17.0	16.9	.951
Partner had a miscarriage	8.8	8.5	.871	8.8	9.7	.505
Partner had a termination	3.0	2.5	.613	1.9	1.8	.853
Partner became pregnant	43.0	46.6	.194	46.6	52.1	.**022**
Numbers studied	1013	481		571	1826	

[Percentages concern the proportion of men in the LOC category who had the history]

In bold are P values of P < .05.

## 4 Discussion

### 4.1 LOC between spouses and over time

This study is among the few that has obtained LOC scores from a large representative population composed of parental dyads at two time points, but is unique because the initial test took place prenatally, and the second six years later Hajek and Konig (2017) collected LOC information from a large representative cohort of adults over a five year period from 2005 to 2010, but did not delineate where participants were in regards to family or examined factors that were associated with changes in LOC. On the other hand, Schneewind (1997) did focus on LOC in parents of children who were first tested when children were aged 10 and tested again 16 years later. However his participants were a select group of nearly two hundred family members whose initial LOC test was not obtained before the child was born. By obtaining mother and father LOC scores prenatally we have the unique advantage of being able to describe parents' LOC perspectives before the arrival of the child and to evaluate the future impact of their prenatal orientations on their own and their children's behavior. Studies have already begun to show the benefit of this approach by revealing that the degree of externality in the parent dyad was associated with (1) negative outcomes in children's eating, sleeping and emotion regulation behaviors during their first 5 years of life (Nowicki, Iles-Caven, Gregory, Ellis, & Golding, 2017a) and (2) the number of teacher-rated emotional and behavioral difficulties when children were 9 and 11 years old (Nowicki, Iles-Caven, Gregory, Ellis, & Golding, 2017b).

The design of the ALSPAC study and the data set it produced not only allowed us to evaluate the associations between parent prenatal LOC and child outcomes but also (1) to gather heretofore nonexistent normative data regarding the nature and stability of the spouses' LOC within their dyads prior to their child's birth and 6 years later, as well as (2) to obtain indicators of the stability of adult male and female LOC scores from before a child was born to a time 6 years later. We know of no other study that has obtained extensive normative LOC information from a large and representative population of adult women and men who were in a relationship with one another over time.

Our results favor the similarity prediction (Byrne, 1969), but just barely. Correlations were positive but low at both testing times. While mothers and their partners tend to share a similar LOC perspective, it is only that, a tendency, and the reality is that for most parent dyads, various LOC combinations exist. To examine associations that might arise from different combinations with child outcomes, Nowicki et al. (2017a) created four combinations of prenatal LOC; internal/internal; mother internal/father external; mother external/father internal; and external/external. They found that as externality increased in the parental dyad so did associations with negative children's adjustment outcomes.

### 4.2 Associations with stability and change in LOC over six years

Moderate positive correlations between pre- and post-test scores indicate that many individuals were changing their LOC orientation over time. We predicted and found that LOC changes, especially toward externality, were associated with *lifestyle*, *social*, and *stressful event* variables. What is clear from the findings is that greater stress and less stability in personal relationships, health, and financial matters are related to remaining or becoming external for both mothers and their partners. In terms of relationships perhaps none are more important than the one between the parents themselves. Externality was associated with stressful spousal issues in the relationship including physical or emotional abuse of one another or other indicators of relationship failure such as divorce, trouble with the law or rejection of the child. Although mothers had more relationship stressors associated with externality than their partners, the direction of the association was the same. Besides their own relationship difficulties being related to externality, both parents indicated that “arguing with family and friends” added to the potential to remain or become external. If mothers and their partners didn't get along then an inability to relate to family and friends would probably increase the likelihood of being associated with feelings of externality.

The importance of the spousal relationship was further substantiated by the finding that living together or living apart was related to stability and change in LOC. External mothers were more likely to move toward internality if they had a live-in partner in contrast to prenatally internal mothers who, if they did not have a live-in partner, moved toward externality; the same association was found for mothers' partners as well.

Financial matters and “work” were also associated with externality for both mothers and their partners. For mothers, externality was associated with “having major financial problems” and “not going back to work” while for men it was “losing a job”, “reduction of income”, and “partner starting new job”. “Work” may be especially important for men's LOC as suggested by the finding that “starting a new job” was the only event associated with internality for them.

Illness was associated with LOC differently for mothers and their partners. For men, “having been very ill himself” was significantly related to *externality* while for women “having a partner who was very ill” was associated with *internality*. In this regard, it is noteworthy that the four events endorsed by women who stayed internal (having had a partner who was very ill; having had a partner who had problems at work; having to go back to work herself; and having problems at work) involved problems in their spousal and work relationships. Perhaps meeting challenges and responding successfully to them facilitated internality in women. Unfortunately, we do not have access to what exactly the problems were or what the women thought, felt and did while experiencing them, but we might learn much from interviewing such women to obtain this information.

Lifestyle differences were also related to LOC stability and change. Clearly, smoking was a significant marker of externality in the ALSPAC population. As smoking increased so did externality. While we cannot establish cause and effect with our design, the fact that smoking is associated with externality and externality in turn is associated with negative outcomes in personality, health, achievement, and adjustment suggests it would be worthwhile having a closer look at how those variables affect one another. Past research suggested a connection between smoking and LOC (Wallston & Wallston, 1978). Because of the association between smoking and externality, it might be helpful to see if stop-smoking programs had any effect on participants' LOC. Unfortunately, research has focused on determining who is more likely to successfully complete a stop-smoking program; not surprisingly most often it is the internals and not the externals (e.g. Kaplan & Cowles, 1978). One potential way around the fact that externals do not respond as well to more general smoking cessation programs is to use individualized interventions favoring those with external as opposed to internal control expectancies (e.g. Steffy, 1975) to see if externals who were successful changed toward internality.

Having a choice about where we live is important, so it is not surprising that ALSPAC participants who had less choice of where to live and found their only choice was to live in public housing rather than renting or owning other dwellings are more external. To offset a lack of choice in where to live, it might be useful to give those in public housing opportunities to exercise some “control” over their living situation. Researchers have found that institutionalized individuals who were given control over small decisions in their lives responded by changing toward internality (e.g. Aasen, 1987). Perhaps pilot programs in which housing authority personnel and those living in public housing could work together to generate lists of ways in which public housing occupants would have the power to make more of their own choices; this might lead to changes toward internality over time.

### 4.3 Strengths and limitations

Among the strengths of this study are the following: (i) it is based on a whole population, defined geographically and consequently not biased by particular selection (e.g. college students). Comparison with census data from 1991 show that the study participants are broadly representative (Fraser et al., 2013). (ii) The numbers considered are far larger than in any other study of LOC over time. (iii) Data on life events were obtained at times between the measures of the LOC orientation of the parents, and consequently cannot have been influenced by the LOC response. (iv) It is the only study to be able to compare the pairs of parents across time with respect to their LOC orientation. The disadvantages concern: (1) the attrition rate in this study, which was particularly high for the mothers' partners; (2) other modes of statistical analysis, choice of confounders and/or unobserved time-constant variables may provide different results; and (3) the fact that we are unable to confirm our results by comparing results with another study, since there is no (to our knowledge) study that has similar longitudinal data available. In consequence these results should be considered as hypothesis generators. Hopefully they will generate further studies over time.

## 5 Conclusions

Thousands of studies confirm the importance of LOC, yet we have learned little from them about true stability and/or change of LOC over time or what is associated with change when it occurs. The present study provides data to begin to better understand LOC, although it is important to note the paternal attrition over time. We found that: (1) LOC in parent dyads is correlated in a low, positive manner; (2) adult LOC is moderately correlated across time; (3) women become more internal from pregnancy to motherhood, whereas their partners become slightly more external; and (4) greater stress especially in relationships, finances, and health, is associated with increasing externality. Future designs need to build in techniques to gather information that would allow for the assessment of the short-term changes that may have been obscured by adaptation/habituation processes over a 6-year span. In this way we can gather additional information to help us to eventually identify variables that may be linked to change in LOC orientations. Hopefully our findings will help to elucidate the role of stressors in the development of internal and external LOC and bring us closer to being able to construct intervention programs to foster the development of appropriate internality, especially in expectant parents.

The following is the supplementary data related to this article.

Supplementary Table 1 Availability of parental LOC data in pregnancy for whole sample and for the parents 6 years after the baby was born, showing distributions of background factors. Supplementary Table 1

## References

[bb0005] Aasen N. (1987). Interventions to facilitate personal control. Journal of Gerontological Nursing.

[bb0010] Barnett B.H., Hanna E.W., Parker G. (1983). Life events for obstetric groups. Journal of Psychosomatic Research.

[bb0015] Birmingham K. (2018). Pioneering ethics in a longitudinal study: The early development of the ALSPAC Ethics and Law committee.

[bb0020] Boyd A., Golding J., Macleod J., Lawlor D.A., Fraser A., Henderson J., Davey Smith G. (2013). Cohort profile: The ‘children of the 90s’—The index offspring of the Avon longitudinal study of parents and children. International Journal of Epidemiology.

[bb0025] Brown G.W., Harris T. (1978). Social origins of depression: A study of psychiatric disorder in women.

[bb0030] Byrne D. (1969). Attitudes and attraction. Advances in Experimental Social Psychology.

[bb0035] Cheng C., Cheung S., Chio J.H., Chan M.S. (2013). Cultural meaning of perceived control: A meta-analysis of locus of control and psychological symptoms across 18 cultural regions. Psychological Bulletin.

[bb0040] Conell-Price L., Jamison J. (2015). Predicting health behaviors with economic preferences and locus of control. Journal of Behavioral and Experimental Economics.

[bb0045] Flouri E. (2006). Parental interest in children's education, children's self-esteem and locus of control, and later educational attainment: Twenty-six-year follow-up of the 1970 British Birth Cohort. British Journal of Educational Psychology.

[bb0050] Fraser A., Macdonald-Wallis C., Tilling K., Boyd A., Golding J., Davey Smith G., Lawlor D.A. (2013). Cohort profile: The Avon Longitudinal Study of Parents and Children: ALSPAC mothers' cohort. International Journal of Epidemiology.

[bb0055] Golding J., ALSPAC Study Team (2004). The Avon Longitudinal Study of Parents and Children (ALSPAC) – Study design and collaborative opportunities. European Journal of Endocrinology.

[bb0060] Golding J., Ellis G., Iles-Caven Y., Gregory S., Nowicki S. (2017). The antecedents of men's locus of control.

[bb0065] Golding J., Gregory S., Iles-Caven Y., Nowicki S. (2017). The antecedents of women's external locus of control: II. Associations with characteristics of her parents and of late childhood. Wellcome Open Research.

[bb0070] Golding J., Iles-Caven Y., Gregory S., Nowicki S. (2017). The antecedents of women's external locus of control: Associations with characteristics of her parents and of early childhood. Heliyon.

[bb0075] Hajek A., Konig H.-H. (2017). Locus of control and frequency of physician visits: Results of a population-based longitudinal study in Germany. British Journal of Health Psychology.

[bb0080] Infurna F.J., Mayer A. (2015). The effects of constraints and mastery on mental and physical health: Conceptual and methodological considerations. Psychology and Aging.

[bb0085] Infurna F.J., Okun M.A. (2015). Antecedents and outcomes of level and rates of change in perceived control: The moderating role of age. Developmental Psychology.

[bb0090] Judge T.A., Bono T.A. (2001). Relationship of core self-evaluation traits-self-esteem, generalized self-efficacy, locus of control and emotional stability-with job satisfaction and job performance. Journal of Applied Psychology.

[bb0095] Kaplan G.D., Cowles A. (1978). Health locus of control and health value in the prediction of smoking reduction. Health Education & Behavior.

[bb0100] Kiesler D. (1982). The 1982 interpersonal circle: A taxonomy for complementarity in human transactions. Psychological Review.

[bb0105] Kormanik M., Rocco T. (2009). Internal versus external control of reinforcement: A review of the locus of control construct. Human Resource Development Review.

[bb0110] Lachman M.E., Weaver S.L. (1998). The sense of control as a moderator of social class differences in health and well-being. Journal of Personality and Social Psychology.

[bb0115] Lefcourt H. (1976). Locus of control. Current trends in theory and research.

[bb0120] Nowicki S. (2016). Choice or chance.

[bb0125] Nowicki S., Duke M.P. (1974). A locus of control scale for college as well as non-college adults. Journal of Personality Assessment.

[bb0130] Nowicki S., Duke M.P., Infurna F., Reich J.W. (2016). Foundations of locus of control research. Perceived control: Theory, research and practice in the first 50 years.

[bb0135] Nowicki S., Iles-Caven Y., Gregory S., Ellis G., Golding J. (2017). The association of prenatal parental locus of control on children's psychological outcomes in infancy and early childhood: A prospective 5year-study. Frontiers in Psychology, Developmental Psychology.

[bb0140] Nowicki S., Iles-Caven Y., Gregory S., Ellis G., Golding J. (2017). The association of prenatal parent locus of control on children's strengths and difficulties in school at year 3 and 6.

[bb0145] Peterson C., Seligman M.E. (1983). Learned helplessness and victimization. Journal of Social Issues.

[bb0150] Peterson C., Stunkard A.J. (1992). Cognates of personal control: Locus of control, self-efficacy, and explanatory style. Applied and Preventive Psychology.

[bb0155] Reich J.W., Infurna F.J. (2016). Perceived control. Theory, research and practice in the first 50 years.

[bb0160] Rotter J. (1954). Social learning and clinical psychology.

[bb0165] Rotter J. (1966). Generalized expectancies for internal versus external control of reinforcement. Psychological Monographs.

[bb0170] Schneewind K., Ruppert J.H., Harrow J. (1998). Personality and family development: An intergenerational longitudinal comparison.

[bb0175] Schneewind K.A. (1997). The intergenerational transmission of locus of control: A 16 year longitudinal study. International conference on “Dynamics of Parenting”.

[bb0180] Seligman M.E.P. (1975). Helplessness: On depression, development and death.

[bb6000] Skinner E.A. (1996). A guide to constructs of locus of control. Journal of Personality and Social Psychology.

[bb0185] Sørlie T., Sexton H.C. (2004). Predictors of change in health locus of control following surgical treatment. Personality and Individual Differences.

[bb0195] Standard Occupational Classification (1990). London: Office of population censuses and surveys.

[bb0200] Stanley F. (1988). Measures of psychosocial variables for the pregnancy home visiting program.

[bb0205] Steffy R.A. (1975). Smoking modification procedure for internal and external locus of control clients. Canadian Journal of Behavioral Science.

[bb6050] Turiano N.A., Chapman B.P., Agrigoroaei S., Infurna F.J., Lachman M. (2014). Perceived control reduces mortality risk at low, not high, education levels. Health Psychology.

[bb0210] Wallston B.S., Wallston K.A. (1978). Locus of control and health: A review of the literature. Health Education Monographs.

[bb0215] Wallston K.A., Wallston B.S., DeVellis R. (1978). Development of the multidimensional health locus of control scales. Health Education Monographs.

