# Supplementary material for: Events associated with stability and change in adult locus of control orientation over a six-year period
Source: Pers Individ Dif. 2018 May 1;126:85–92. doi: 10.1016/j.paid.2018.01.017 (PMC5818169; doi:10.1016/j.paid.2018.01.017)
Supplement: Supplementary Table 1 — Availability of parental LOC data in pregnancy for whole sample and for the parents 6 years after the baby was born, showing distributions of background factors. [file mmc1.docx]

**Supplementary Table**

Supplementary Table 1. Availability of parental LOC data in pregnancy for whole sample and for the parents 6 years after the baby was born, showing distributions of background factors.

| **Features measured in pregnancy** | **Mothers in pregnancy** | **Maternal LOC after 6 years** | **Paternal LOC after 6 years** |
| --- | --- | --- | --- |
| Housing tenure |  |  |  |
| Owned/mortgaged | 73.2% | 81.1% | 85.0% |
| Publically owned | 14.4% | 9.2% | 6.5% |
| Other | 12.4% | 9.7% | 8.5% |
|  | (n= 13,346) | (n=8,308) | (n=4,372) |
| Age of mother at conception |  |  |  |
| < 20 | 6.6% | 3.2% | 1.9% |
| 20-24 | 22.3% | 18.0% | 15.6% |
| 25-34 | 65.6% | 71.1% | 72.9% |
| 35+ | 5.5% | 8.7% | 9.6% |
|  | (n=14,532) | (n=8,526) | (n=4,458) |
|  |  |  |  |
| Social class (partner’s occupation) |  |  |  |
| Non-manual | 55.6% | 59.5% | 66.9% |
| Manual | 44.4% | 40.5% | 33.1% |
|  | (n = 10,618) | (n=7,578) | (n=4,105) |
|  |  |  |  |
| Maternal education level |  |  |  |
| <O-level | 25.4% | 23.8% | 19.4% |
| O-level | 36.9% | 35.7% | 33.7% |
| >O-level | 37.7% | 40.5% | 47.9% |
|  | (n = 12,370) | (n=8,291) | (n=4,362) |
|  |  |  |  |
| Mother’s parity |  |  |  |
| 0 | 44.9% | 46.0% | 47.8% |
| 1 | 34.8% | 35.5% | 34.7% |
| 2+ | 20.3% | 18.5% | 17.5% |
|  | (n = 12,971) | (n=8,279) | (n=4,365) |
|  |  |  |  |
| Parent was smoking mid-pregnancy |  |  |  |
| Yes | 19.6% | 16.0% | 12.1% |
| No | 80.4% | 84.0% | 87.9% |
|  | (n = 13,274) | (n=8,394) | (n=4,412) |
|  |  |  |  |
